# Impact of a postgraduate year one (PGY-1) otolaryngology bootcamp on procedural skill development

**DOI:** 10.12688/mep.19187.1

**Published:** 2022-07-04

**Authors:** Benjamin Wajsberg, Daniel Li, Avraham Kohanzadeh, Anna C. Bitners, Scott Gorthey, Marc J. Gibber, Esther Rong, John P. Bent, Mona Gangar, Christina J. Yang

**Affiliations:** 1 Albert Einstein College of Medicine, Bronx, New York, USA; 2 Department of Surgery (Division of Otolaryngology), Yale School of Medicine, New Haven, Connecticut, USA; 3 Department of Pediatrics, Johns Hopkins Medicine, Baltimore, Maryland, USA; 4 Department of Otorhinolaryngology–Head & Neck Surgery, Montefiore Medical Center, Bronx, New York, USA; 5 Department of Radiology, Montefiore Medical Center, Bronx, New York, USA; 6 Department of Pediatrics, Montefiore Medical Center/Children’s Hospital at Montefiore, Bronx, New York, USA

**Keywords:** Bootcamp, Medical education, Otolaryngology, Procedural skills

## Abstract

**Background:** To measure the impact of an intensive eight-week postgraduate year one (PGY-1) otolaryngology bootcamp on the acquisition and retention of otolaryngology residents’ procedural skills compared to the traditional method of skill acquisition through clinical exposure.

**Methods: **Residents at our institution were evaluated on their performance of flexible laryngoscopy, suture ligature, and rigid bronchoscopy setup at three time points: pre-bootcamp, one-week post-bootcamp, and one-year post-bootcamp. Video recordings were scored by two blinded faculty reviewers using a multipoint rating system. A control group of rising postgraduate year two (PGY-2) residents who did not participate in bootcamp were recorded performing these same skills. Scores in the three skills were compared between groups via *t*-tests. The eight-week bootcamp curriculum for PGY-1s was held at the Montefiore Einstein Center for Innovation in Simulation at Albert Einstein College of Medicine/Montefiore Medical Center. The participants were two classes of PGY-1 residents (n=8) at our institution who participated in a bootcamp at the beginning of residency, and one class of rising PGY-2 residents (n=3) who did not participate in a bootcamp (control group).

**Results: **A comparison of pre-bootcamp scores to one-week post-bootcamp scores showed significant improvement in suture ligature ( *P*<0.05) and rigid bronchoscopy ( *P*<0.05), but no difference in flexible laryngoscopy ( *P*=0.54). Suture ligature ( *P*=0.09) and rigid bronchoscopy ( *P*=0.25) skills were not significantly different from one-week post-bootcamp to one-year post-bootcamp; however, a significant skill improvement was observed in flexible laryngoscopy ( *P*<0.05). By June of PGY1 year, the two bootcamp cohorts were similar to controls in all three skills: flexible laryngoscopy ( *P*=0.05), rigid bronchoscopy ( *P*=0.26), and suture ligature ( *P*=0.10).

**Conclusions: **Participation in PGY-1 bootcamp was associated with improved acquisition and short-term retention of basic procedural skills, suggesting that bootcamps can be an effective arena to teach basic skills in otolaryngology. PGY-1 bootcamp is a promising arena for multi-institutional development.

## Introduction

Simulation is a powerful and increasingly popular tool for medical resident education and training with the ultimate goal of improving patient safety. Simulation is widely used in otolaryngology, with 82% of otolaryngology residency programs reporting some use of simulation at their institution ^1^.

In the field of otolaryngology, several institutions have developed simulation-based bootcamps for resident education. Bootcamps are defined as short, intensive training sessions (usually one to three days), which teach or reinforce technical skills, clinical knowledge, and team dynamics ^2– 5^. The immediate purpose of bootcamp is to increase residents’ comfort and competence with basic knowledge and technical skills relevant to the field of otolaryngology with the goal of improving patient safety. Bootcamps have demonstrated success in increasing resident confidence ^6^ and training efficiency ^7^, though their impact on the development of technical skills is underexplored.

Our institution developed a unique eight-week bootcamp curriculum for postgraduate year one’s (PGY-1) to jumpstart their education at the beginning of residency. It is novel in the extended timeline of eight weeks and the evaluation of task-based skills as an outcome metric. We designed and implemented the extended bootcamp experience with goals of (1) increasing competency in basic procedural skills, (2) improving proficiency in ENT emergencies, and (3) expanding basic otolaryngology knowledge. We sought to objectively evaluate resident technical ability before and after participation, hypothesizing that scores in flexible laryngoscopy, suture ligature, and rigid bronchoscopy set-up would increase after bootcamp and be retained one year later. We also hypothesized that residents at the beginning of postgraduate year two (PGY-2) who had participated in bootcamp as PGY-1s would demonstrate better technical skills than a control group of early PGY-2 residents who had not participated in bootcamp.

**Meeting information:** This work was presented at the Triological Society Combined Otolaryngology Spring Meeting, Austin, Texas, May 4, 2019.

## Methods

### Ethical statement

The study was approved by the Einstein Institutional Review Board (2018-9818). Resident participation in the study was voluntary and all participants provided written informed consent.

### Curriculum design and programming

Faculty and senior residents in the Otolaryngology—Head and Neck Surgery Department at Montefiore Medical Center contributed to curriculum design. Bootcamp took place over eight weeks during the first summer of residency at the Montefiore Einstein Center for Innovation in Simulation at Albert Einstein College of Medicine/Montefiore Medical Center in Bronx, NY. Modules held daily from 8am to 12pm incorporated multimodal learning techniques including lectures, task trainer stations, simulated scenarios, and debriefing sessions. The last two days were dedicated to evaluation in the form of a written test and skills assessment. The bootcamp has since been redesigned into weekly sessions held on Friday mornings during the first two months of the year. The entire curriculum of bootcamp is beyond the scope of this paper and has been previously described by our group ^8^. Here we focus on the assessment of procedural skill acquisition and retention as evaluated with three task trainer modules: flexible laryngoscopy, suture ligature, and rigid bronchoscopy set-up. A total of eight PGY-1 members from two separate years participated. The study started in June 2016 and ran through August 2019.

### Faculty and resident educators

Two faculty members (MJG, CJY) taught the majority of modules, with assistance from senior (postgraduate year four [PGY-4] and postgraduate year five [PGY-5]) residents.

### Task trainers

We chose three tasks which incorporate fundamental skills in otolaryngology including airway evaluation (flexible laryngoscopy), hemorrhage control (suture ligature), and airway intervention (rigid bronchoscopy). We chose these procedures because they are fundamental to our field and incorporate a variety of skills including visuospatial ability, dexterity, and memorization.

***Flexible laryngoscopy.*** In this module, participants performed flexible laryngoscopy on a mannequin using a 3.5 mm flexible laryngoscope with an attached Stryker HD camera and light source. The image was displayed on a Storz camera tower during the procedure.

***Suture ligature.*** Participants were provided a variety of instruments with which to perform suture ligature of a mock blood vessel represented by a 16 French red rubber catheter.

***Rigid bronchoscopy setup.*** This module challenged participants to assemble an array of rigid bronchoscope parts into proper functioning format. 

### Filming

Bootcamp participants were filmed at three time points: before beginning bootcamp (in the first week of intern year), one week after completing bootcamp (in September of intern year), and one year after bootcamp (in the first weeks of PGY-2 year). During filming sessions, one resident at a time entered the room and was filmed with an iPad while they performed each task.

### Faculty rating

Blinded faculty (MG, JPB) reviewed and rated videos of the residents’ performance in each task. To ensure that faculty were properly blinded, identification badges were removed prior to filming, residents wore identical gloves and hospital-issued scrubs, and the video recordings restricted the field to the participant’s hands and removed sound. To limit bias, pre-, post-, and one-year recordings were randomized for rating in a single batch. The rating scale for flexible laryngoscopy was based on a previously published assessment tool ^9^. Items which were not applicable to a mannequin (as opposed to a human patient) were removed, including obtaining consent, patient positioning, and application of local anesthesia ( Table 1). Assessment tools for the other two tasks were based on the rating system for flexible laryngoscopy and were intended to assess the performer in a stepwise approach and to judge the overall performance in the task. Each task was broken down into core components and each component was scored on a scale from 1 to 5 ( Table 1). The scores for all components were averaged to create a composite score for each task that was used for statistical evaluation.

**Table 1.** A rating tool for the three tasks was completed by faculty reviewers.

Instructions:	
For each item, please rate the resident's ability on a scale from 1 to 5. 1: Unable to perform 3: Performs with minimal prompting (Competent) 5: Performs easily with good flow (Expert)	
Flexible Laryngoscopy	Score
Introduction of scope into nose (correct orientation)	
If camera/scope is not oriented correctly, is corrective action taken?	
Advancement to the soft palate	
Assessment of the pharynx	
Assessment of the larynx	
Suture Ligature	Score
Places suture through the vessel	
Asks assistant to release hemostat after the first knot is placed	
At the end of the procedure, is there at least one knot around the entire vessel?	
Rigid Bronchoscope Setup	Score
Light cord attached correctly to telescope	
Telescope fully locked into the bronchoscope	
All pieces of the bronchoscope attached	

### Control group

Three PGY-2 residents who had not participated in bootcamp as PGY-1s composed the control group. (The fourth resident in that class was completing an off-site rotation and was unable to participate.) The control group’s only evaluation and the study group’s last evaluation both occurred in the first week of PGY-2 year such that they had completed the same amount of residency training time.

### Statistical analysis

Bootcamp participants’ performance in flexible laryngoscopy, suture ligature, and rigid bronchoscopy at the various time points were compared using paired *t*-tests. Unpaired *t*-tests were used to compare the intervention group with the control group. GraphPad Prism 9.3.1 (company located in San Diego, CA) was used for statistical analysis. In all cases, significance was defined as alpha = 0.05.

## Results

**Figure 1.**
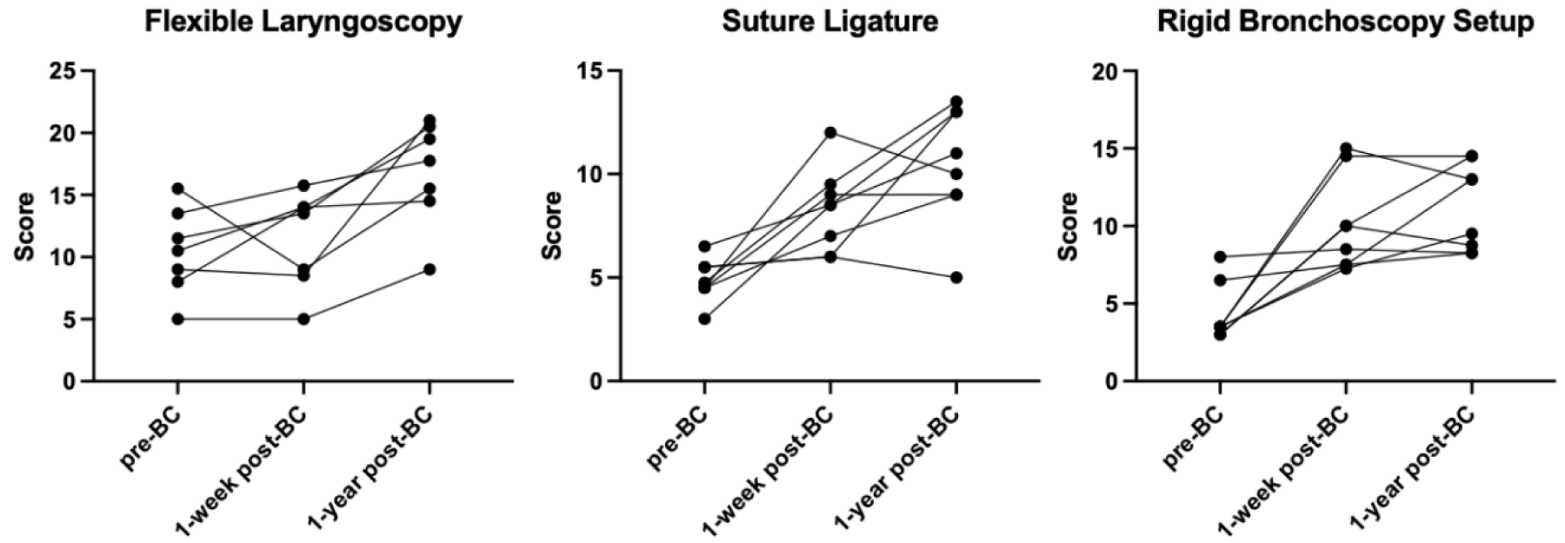
Plot of the residents’ scores pre-bootcamp, one-week post-bootcamp, and one-year post-bootcamp. BC = bootcamp.

Eight PGY-1 residents who participated in this bootcamp were evaluated in flexible laryngoscopy, suture ligature, and rigid bronchoscopy setup at three time points: pre-bootcamp, one-week post-bootcamp, and one-year post-bootcamp. One resident was not evaluated in flexible laryngoscopy one week after participation in bootcamp and thus was excluded from the analysis for that task. Each individual’s scores over time are displayed in Figure 1. The mean scores for each task at each time point are displayed in Table 2. The combined mean score for each task increased after bootcamp and during the following year. Statistical significance was determined for three comparisons ( Table 2): (1) bootcamp participants before and one week after course completion, (2) bootcamp participants one week after and one year following course completion, and (3) bootcamp participants one year after completion compared to the control group.

**Table 2.** Data is presented for three comparisons: pre-bootcamp vs one-week post-bootcamp, one-week post-bootcamp vs one-year post-bootcamp, and one-year post-bootcamp vs one-year without bootcamp (control group). FL = flexible laryngoscopy, SL = suture ligature, RB = rigid bronchoscopy set up, BC = bootcamp.

	Mean (SD)		*P*-value	Mean (SD)		*P*-value	Mean (SD)		*P*-value
	pre-BC	1-week post-BC		1-week post-BC	1-year post-BC		1-year post-BC	1-year non-BC	
**FL**	10.43 (3.25)	11.39 (3.62)	0.54	11.39 (3.62)	16.82 (3.92)	**0.01**	16.82 (3.92)	10.67 (2.32)	0.05
**SL**	4.84 (0.96)	8.31 (1.87)	**0.006**	8.31 (1.87)	10.44 (2.66)	0.09	10.44 (2.66)	7.00 (2.12)	0.10
**RB**	4.31 (1.75)	10.03 (2.91)	**0.006**	10.03 (2.91)	11.22 (2.61)	0.25	11.22 (2.61)	8.83 (2.66)	0.26

**Figure 2.**
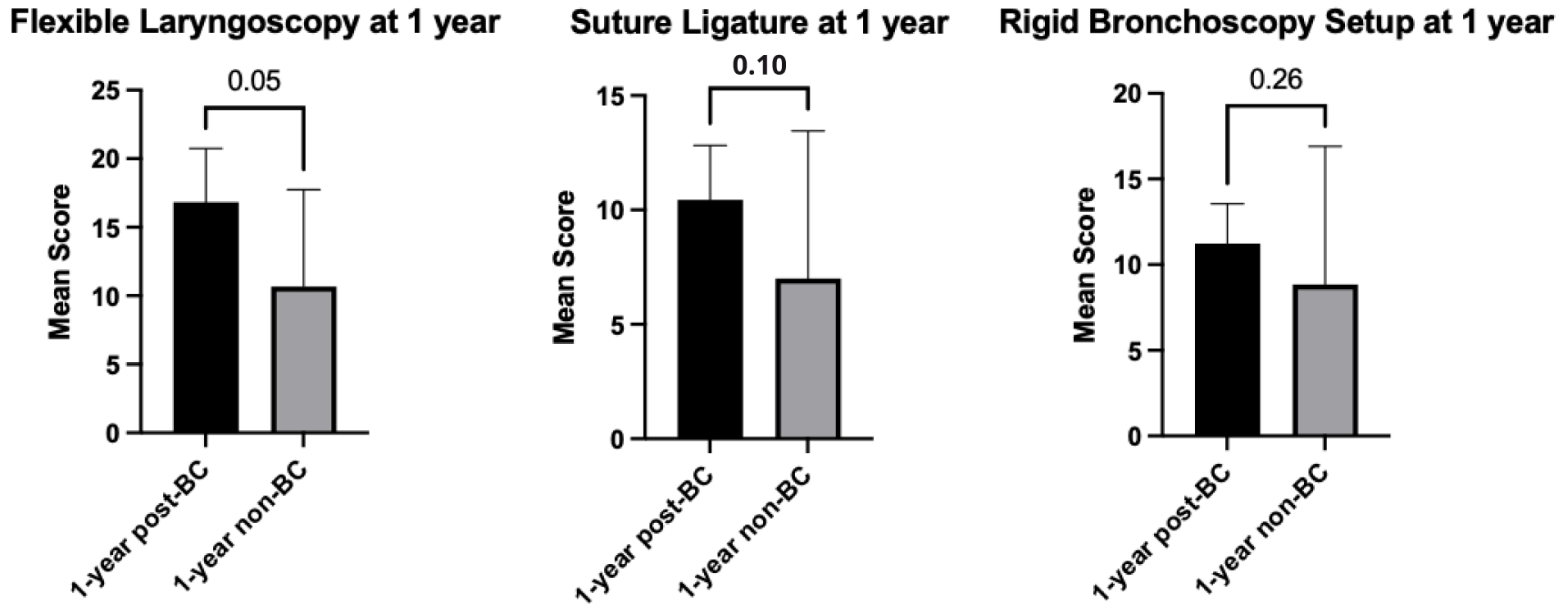
Plot comparing residents’ scores one-year post-bootcamp and one-year without bootcamp (control group). BC = bootcamp.

A comparison of pre-bootcamp scores to one-week post-bootcamp scores showed significant improvement in suture ligature ( *P*<0.05) and rigid bronchoscopy ( *P*<0.05), but no difference in flexible laryngoscopy ( *P*=0.54). Suture ligature ( *P*=0.09) and rigid bronchoscopy ( *P*=0.25) skills were not significantly different from one-week post-bootcamp to one-year post-bootcamp; however, a significant skill improvement was observed in flexible laryngoscopy ( *P*<0.05). By June of PGY1 year, the two bootcamp cohorts were similar to controls in all three skills: flexible laryngoscopy ( *P*=0.05), rigid bronchoscopy ( *P*=0.26), and suture ligature ( *P*=0.10) ( Figure 2).

## Discussion

Although previous studies have demonstrated improvement in participants’ confidence and knowledge after participation in otolaryngology bootcamps ^2, 10– 12^, they are limited by their reliance on survey-based self-reporting over objective, blinded metrics. Of note, some studies have started to provide objective evidence that bootcamps increase knowledge as demonstrated by improved scores on multiple-choice exams ^13, 14^ and better performance in simulated scenarios ^13^. Our study is unique in its focus on the objective evaluation of the impact of an otolaryngology bootcamp on procedural skill acquisition and retention via the use of blinded faculty rating.

We found that residents who participated in bootcamp significantly improved in suture ligature and rigid bronchoscopy setup skills immediately after participation in the bootcamp, which suggests that bootcamps can improve objectively measured technical skills. Additionally, residents demonstrated retention of these skills at the one-year mark, and scores were similar to controls. Bootcamps participants’ flexible laryngoscopy skills started at a higher level at baseline, which might in part explain the lack of significant improvement immediately after bootcamp. It is likely that PGY-1 residents were more commonly exposed to flexible laryngoscopy than suture ligature and rigid bronchoscopy setup in their usual clinical work over the course of their PGY-1 year, which might explain why flexible laryngoscopy skills continued to improve from immediate post-bootcamp to the one-year mark compared to the other skills.

 Our experience suggests that, beyond increasing resident self-confidence and knowledge, participation in bootcamp can accelerate acquisition of technical skills in otolaryngology compared to clinical experience alone. This finding supports the increased use of simulation-based bootcamps for resident education in the field of otolaryngology. This simulation education study targeted to junior residents focused on fundamental manual skills in otolaryngology, consistent with junior residents’ preference for procedural skills and didactic teaching over non-technical skills such as communication and leadership at this stage in their training ^12^. As residents learn increasingly complex operative skills and techniques over the course of residency, simulation-based learning for senior residents should incorporate more advanced skills and focus on the non-technical aspects of patient management and teamwork.

The main limitation of our study is the small sample size inherent in studying single institution surgical specialty residency programs. Additionally, the control group was comprised of rising PGY-2s who spent three months on a dedicated otolaryngology service whereas the PGY-1s who participated in bootcamp spent six months on a dedicated otolaryngology service, which may account for some of the improvement in their scores of the course of the year but would be unlikely to influence the immediate post-bootcamp results. Furthermore, familiarity prior to training or clinical experience with the skills tested could not be controlled for within each cohort. The fidelity of mannequins in lieu of patients for flexible laryngoscopy and suture ligature skills, though standard for training, was also a limitation. The rigidity of the rubber substituting for mucosa may have limited the ability of participants to display proper flexible laryngoscopy technique and could partially explain the greater variability in performance observed for that task, although it is unlikely to be different by observation timepoint. As a resident-driven program, our institution’s bootcamp evolves each year as we tailor it to our residents’ perceived areas of need. More focused study of the skills valued by residents and program directors will allow us to further develop the program to address areas for further growth and attention.

Future investigation is needed to explore whether task acquisition during bootcamp correlates to improved resident performance and patient safety, overall speed of surgical skill acquisition and fluency, and improved patient care.

## Conclusion

Simulation-based bootcamp can be an effective method to teach fundamental procedural skills in the field of otolaryngology and is a promising arena for multi-institutional developments.

## Data availability

### Underlying data

B2Share: Impact of PGY-1 Otolaryngology Bootcamp on Procedural Skill Development

http://doi.org/10.23728/b2share.c6b965776b5844fb80046f1b07da971b ^15^

This project contains the following underlying data:

- Flexible Laryngoscopy A-E.csv (raw data of participants)
- Rigi Bronchoscopy A-C.csv (raw data of participants)
- Suture Ligature A-C.csv (raw data of participants)

Data are available under the terms of the Creative Commons Zero “No rights reserved” data waiver (CC0 1.0 Public domain dedication).
