# Supplementary material for: Impact of a postgraduate year one (PGY-1) otolaryngology bootcamp on procedural skill development
Source: MedEdPublish (2016). 2022 Jul 4;12:47. [Version 1] doi: 10.12688/mep.19187.1 (PMC9427082; doi:10.12688/mep.19187.1)
Supplement: Peer review history

# Peer review: Reviewer response for version 1

**DOI:** 10.21956/mep.20556.r32390

**Published:** 2022-08-10

**Authors:** Gene C. Liu

**Affiliations:** 1 Division of Otolaryngology - Head and Neck Surgery, Cedars-Sinai Medical Center, Los Angeles, CA, USA

Simulation in technical education and Bootcamps have gained traction in resident education.  The efficacy of such teaching methods are still unknown and the authors address an important issue in an organized and thoughtful manner. 

 There is clear acknowledgment of the limitations of the study, appropriate analysis of the data, clear and organized methods. There are no significant concerns regarding this submission.

Have any limitations of the research been acknowledged?

Yes

Is the work clearly and accurately presented and does it cite the current literature?

Yes

If applicable, is the statistical analysis and its interpretation appropriate?

Yes

Are all the source data underlying the results available to ensure full reproducibility?

Yes

Is the study design appropriate and is the work technically sound?

Yes

Are the conclusions drawn adequately supported by the results?

Yes

Are sufficient details of methods and analysis provided to allow replication by others?

Yes

Reviewer Expertise:

Simulation Education

I confirm that I have read this submission and believe that I have an appropriate level of expertise to confirm that it is of an acceptable scientific standard.

# Peer review: Reviewer response for version 1

**DOI:** 10.21956/mep.20556.r32336

**Published:** 2022-07-27

**Authors:** James J. Kearney, Kara Silberthau, MD

**Affiliations:** 1 Department of Otorhinolaryngology - Head and Neck Surgery, University of Pennsylvania, Philadelphia, PA, USA; 2 University of Pennsylvania Perelman School of Medicine, Philadelphia, PA, USA

This article attempts to provide something that has been lacking in simulation education, a demonstration that the training actually accelerates the acquisition of skills without endangering patients. The authors should be acknowledged for their dedication to advancing ORL resident education through a very thorough training program and their time spent researching the topic. 

 The methods are sound and reproducible. The number of participants was limited as this is a single institution study. 

 The authors acknowledge that there was heterogeneity in the PGY-2 control group and the PGY-1 experience due to RRC changes. 

 We have no major criticisms and recommend this submission for passing peer review.

Have any limitations of the research been acknowledged?

Yes

Is the work clearly and accurately presented and does it cite the current literature?

Yes

If applicable, is the statistical analysis and its interpretation appropriate?

Yes

Are all the source data underlying the results available to ensure full reproducibility?

Yes

Is the study design appropriate and is the work technically sound?

Yes

Are the conclusions drawn adequately supported by the results?

Yes

Are sufficient details of methods and analysis provided to allow replication by others?

Yes

Reviewer Expertise:

Simulation training for resident education

We confirm that we have read this submission and believe that we have an appropriate level of expertise to confirm that it is of an acceptable scientific standard.
